# Allergenicity reduction of the bio-elicited peanut sprout powder (BPSP) and toxicological acceptance of BPSP-supplemented diets assessed with ICR mice

**DOI:** 10.1007/s13197-022-05537-7

**Published:** 2022-07-10

**Authors:** Brian B.-C. Weng, Yu-Chia Liu, Brittany L. White, Ju-Chun Chang, Jack P. Davis, Shih-Hsuan Hsiao, Robin Y.-Y. Chiou

**Affiliations:** 1 Department of Microbiology, Immunology and Biopharmaceuticals, National Chiayi University, Chiayi, 60004 Taiwan, Republic of China; 2 Department of Food Science, National Chiayi University, Chiayi, 60004 Taiwan, Republic of China; 3 Market Quality and Handling Research Unit, USDA ARS, North Carolina State University, Raleigh, NC 27607 USA; 4 Department of Food, Bioprocessing and Nutrition Sciences, North Carolina State University, Raleigh, NC 27607 USA; 5 Veterinary Diagnostic Laboratory, College of Veterinary Medicine, University of Illinois, Urbana, IL 61802 USA

**Keywords:** Peanut, Allergenicity, BPSP (bio-elicited peanut sprout powder), Stilbenoids, Resverachidins, Food safety assessment, BPSP-supplemented diets

## Abstract

The allergenic and toxicological acceptances of the bio-elicited peanut sprout powder (BPSP) have not been assessed. BPSP was generated from peanut kernels germinated at 26–28 °C for 72 h (designated as 72 h-NGS). The 72 h-NGS were subsequently sliced, incubated, dried, defatted and pulverized to generate bio-elicited peanut sprout powder (BPSP). Protein solubility of BPSP increased 2.6-fold compared to 72 h-NGS. SDS-PAGE analysis revealed BPSP production triggered extensive degradation of the high-molecular weight peanut allergic proteins, mainly Ara h 1 and Ara h 3. Western blotting detected with peanut allergic patients’ IgE indicated decreased in vitro reactivity. Food safety assessment of BPSP was performed with ICR mice fed with basal (control) and three doses of formulated BPSP-supplemented diets containing 0.11 g (normal), 2.5 g (high) and 25 g (super high) BPSP /kg BW. Animals appeared healthy with steady body weight gain in all groups during the entire 35-day dietary intervention. Hematological and serum biochemical analyses revealed no significant difference among groups. Histopathological examination on the tissue sections of primary organs further supported safety with no pathologies. The in vitro allergic reduction and toxicological safety in the BPSP-supplemented dietary intervention in the ICR mice study, support moving forward with BPSP-involved product development.

**Supplementary Information:**

The online version contains supplementary material available at 10.1007/s13197-022-05537-7.

## Introduction

The potential for food induced allergic reactions is a critical food safety concern. Accidental ingestion of peanut allergens in foods containing peanut ingredients by sensitized individuals may cause serious systemic allergic reactions or even fatal food-induced anaphylaxis (Yocum and Khan 1994; Shah et al. 2019). The major peanut allergens, Ara h 1, Ara h 2, and Ara h 3 are seed storage proteins and primary allergens recognized by more than 50% of peanut-allergic patients (Koppelman et al. 2001; Mueller et al. 2014). As estimated, in the USA, 70 to 90% of the allergic patients are reactive to Ara h 1 and Ara h 2 (Burks et al. 1995; Clarke et al. 1998). Accordingly, peanut allergy has not only impacted the affected individuals but the development of peanut related industries (White et al. 2014). Current management mostly relies on allergic individuals avoiding foods containing peanut ingredients, even though a majority of the adverse events occur with accidental ingestion (Shah et al. 2019).

Peanut allergenic proteins are not eliminated or evaporated upon thermal or vacuum-facilitated food processing treatments, including thermal processing, high-pressure, pulsed ultraviolet light, high-intensity ultrasound, irradiation and pulsed electric field treatments (Maleki and Hurlburt 2004; Nesbit et al. 2012; White et al. 2014; Rao et al. 2016). Enzymatic hydrolysis of peanut allergenic proteins could perhaps diminish peanut allergenicity from the chemical and biochemical aspects (Cabanillas et al. 2012; Shi et al. 2012; White et al. 2014; Shah et al. 2019). Cabanillas et al. (2012) reported that hydrolysis of the soluble roasted peanut proteins by Alcalase® (an endoprotease) has decreased IgE reactivity, and was presumed to be better than by Flavourzyme® (an exoprotease). Interestingly, naturally activated indigenous enzymatic hydrolysis during seed germination could be regarded as a novel approach to effectively reduce peanut allergenicity. Previous investigations on temporal and spatial gene expressions of *ara h 1*, *ara h 2* and *ara h 3* have shown alterations during peanut seed germination and seedling growth (Kang et al. 2007). They reported the protein levels of Ara h 1, Ara h 2 and Ara h 3 changed dramatically as affected by germination time and anatomy in the embryonic axes and cotyledons during seedling growth. Therefore, the allergenicity of the soluble proteins extracted from peanut kernels after subjection to germination and followed bio-elicitation in preparation of bio-elicited peanut sprout powder (BPSP) deserves further investigations.

Peanut (*Arachis hypogaea* L.) can be used for the biosynthesis of a wide spectrum of stilbene compounds including resveratrol and varied stilbenoids (Chang et al. 2006; Sobolev 2011; Sobolev et al. 2016; Chiou et al. 2017; Khawand et al. 2018; Cheng et al. 2021). Peanut sprouts have been demonstrated and regarded as a functional food bearing potent health benefits (Wang et al. 2005; Lin et al. 2008; Seo et al. 2014; Ha et al. 2015). After germination, sprouted cotyledons can be intentionally sliced and further processed to prepare BPSP enriched with stilbenoid antioxidants, including resveratrol, arachidin-1, arachidin-3, isopentadienylresveratrol (IPD), arahypin-5 and other newly identified phenolic compounds (Chang et al. 2006; Chiou et al. 2017; Cheng et al. 2021). These bioactive phenolic compounds mainly containing resveratrol derivatives and arachidins, that could be grouped as a family of peanut resverachidins. Previously, BPSP-supplemented feeding experiments in the retired breeders of the inbred Balb/c and outbred ICR mouse strains each lasting for more than 2 years demonstrated longevity extension in mice fed with BPSP-supplemented diets (Chiou et al. 2017). Recently, BPSP-supplemented diets showed effectiveness in inhibition of benign prostatic enlargement in the orchiectomized Sprague–Dawley rats (Cheng et al. 2021).

Along with the emerging diverse perspectives in the use of BPSP for development of nutraceuticals and functional foods, the food toxicological safety consideration, accessible doses and particularly the peanut allergenicity are prerequisites that need to be evaluated. Therefore, in the current study, BPSP extracted soluble protein analysis in allergenicity in vitro and the healthy food toxicological evaluation based on the protocol of the Taiwan Food and Drug Administration, Ministry of Health and Welfare (2010) were conducted and addressed.

## Materials and methods

### Sample preparation for allergenic analysis

#### Preparation of the bio-elicited peanut sprout powder (BPSP)

Sound and graded peanut kernels (*Arachis hypogaea* L., Tainan 11, a Spanish cultivar) (assigned as the raw kernel) were soaked with tap water for 4 h at 26–28 °C for imbibition. After draining, the kernels were incubated 72 h in a growth chamber at 26–28 °C for sprouting (assigned as 72 h normally germinated sprout, 72 h-NGS). The cotyledons from each normal sprouted kernel were collected, sliced and incubated in a bio-reactor as a wounding treatment to activate secondary metabolite biosynthesis for bio-elicitation (Arora and Strange 1991; Chang et al. 2006). The slices were then roasted in an air-forced oven at 120 °C for 30 min, ground into paste and defatted with *n*-hexane to prepare the bio-elicited peanut sprout power (BPSP). For comparison on identical basis, the cotyledons from the raw kernels and 72 h normally germinated sprouts were also manually collected, and cut into slices, evenly roasted at 120 °C for 30 min, ground into paste and defatted with *n*-hexane to prepare raw kernel and 72 h-NGS powders. All powders were sealed in heavy-duty PE-plastic bags and stored at -20 °C until use.

#### Peanut allergen analysis

The powders of the raw kernel, 72 h-NGS and BPSP were dispersed (5%, w/v) in phosphate buffered saline (PBS, pH 7.4) and allowed to shake at room temperature for 20 min. The dispersions were then centrifuged at 5,500 × *g* for 10 min at 10 °C. Each of the supernatants was recovered and subjected to soluble protein quantification by BCA assay with bovine serum albumin (BSA) as a standard (Pierce, Rockford, IL, USA). Varying protein concentrations, namely, 10, 13 and 17.1 µg obtained from 72-NGS and 10, 13, 17.1 and 44.6 µg obtained from BPSP were loaded for SDS-PAGE analysis using the Bio-Rad Criterion system (Bio-Rad Laboratories Inc., Des Plaines, IL, USA). Based on the preliminary trials, approximately 17 µg protein was appropriate and applied in the followed investigation.

For Western blotting, the procedure of Shi et al. (2012) was followed with minor modification. Sample protein (ca. 17 µg) was loaded in each well and subjected to SDS-PAGE analysis as described above. Then, proteins were transferred onto Immobilon ® transfer membranes and stained by Ponceau S solution. The membranes were then blocked with bovine albumin solution Fraction V (Gibco, Life Technologies Corp., Carlsbad, CA.) and incubated overnight with pooled plasma from confirmed peanut allergic patients with IgE levels > 100 kU/L as determined by ImmunoCAP™ method. For comparison, the protein fractions of raw and roasted reference peanut (Georgia Green) were treated concurrently in the same manner. The blots were submerged in SuperSignal ® West Pico Chemiluminescent Substrate, and a Chemi Doc TM Imaging System was used to visualize the blots.

### Food safety assessment on the ICR mice fed with BPSP-supplemented diets

#### Formulation and stilbenoids analysis by HPLC

For HPLC analysis, 0.1 g BPSP was weighed, deposited into a 10 mL-screw tube and homogenized with 5 mL 60% ethanol (Kinematica AG Polytron PT3000, Littau, Switzerland) at 15,000 rpm for 1 min. After heating the crew-capped tube in a water bath at 70 °C for 30 min with occassioal shaking, the tubes were centrifuged (Sigma Labrozentrifugen 2K15, Osterode am Harz, Germany) at 15,000 *g* for 15 min at 20 °C. The supernatant was membrane-filtered (0.45 μm) and stored at 4 °C for HPLC analysis.

For HPLC analysis, the procedure of Chang et al. (2006) was followed with minor modification. A dual pump (L-7100 pump, Hitachi Co., Tokyo, Japan) equipped with an UV-detector (L-7420 UV detector, Hitachi Co., Tokyo, Japan) run with an ODS column (Hypersil ODS column, 250 × 4.60 mm, 5 μm, Thermal Hypersil Ltd., Cheshire, UK). Two solvents, i.e., A: methanol and B: pure water were run with a gradient program initiated by 0 min: 0% A; 10 min: 30% A; 20 min: 100% A; 23 min: 100% A; 28 min: 0% A and 30 min: 0% A. The injection volume and monitoring wavelengh were 20 µL and 254 nm, respectively.

#### Preparation of BPSP-supplemented diets

For diet preparation (Table 1), the formulated powder ingredients excluding vitamins and minerals were premixed and ground with boiling water into a paste and blending in corn oil (Jendah Mixer JD-401, Chiayi, Taiwan). After mixing with vitamins and minerals premix®, the paste was spread and pressed into a sheet onto a mode (30 × 50 × 1.5 cm), then cut into square cubes (1 × 1.5 × 1.5 cm) and subjected to dehydration at 50 °C in an air-forced oven with occasional agitation until constant weights were reached, ranged from 36 to 48 h varied on batch conditions.

**Table 1:** Experiment diets formulated with different levels of bio-elicited peanut sprout powder (BPSP) in substitution of soybean meal were subjected to a 35-days dietary intervention study

Ingredient	Diet composition, g/100 g			
	Control	Normal BPSP	High BPSP	Super-high BPSP
BPSP^a^	0	0.11	2.5	25
Soybean protein	10.0	9.9	7.5	0
Full-fat soybean powder	27.0	27.0	27.0	12
Corn starch	20.0	20.0	20.0	20.0
Fish meal	5.0	5.0	5.0	5.0
Wheat bran	8.0	8.0	8.0	8.0
Sucrose	19.54	19.54	19.54	19.54
Corn oil	4.8	4.8	4.8	4.8
Salt	0.3	0.3	0.3	0.3
Limestone powder	0.29	0.29	0.29	0.29
L-lysine	0.17	0.17	0.17	0.17
DL-methione	0.2	0.2	0.2	0.2
AIN-Vitamin premix^b^	1.0	1.0	1.0	1.0
AIN-Mineral premix^c^	3.5	3.5	3.5	3.5
Chlorine bitartrate	0.2	0.2	0.2	0.2

^a^BPSP: Bio-elicited peanut sprout powder

^b^AIN-93 Vx Vitamin mix (MP Biochemicals, Inc., Irvine, CA, USA)

^c^AIN-93 M mineral Mix (MP Biochemicals, Inc., Irvine, CA, USA)

For different levels of BPSP-supplemented diets preparation, the amount inclusions were determined based on stilbenoid content in BPSP. Stilbenoid content was obtained by calculated specific peaks of the area shown in HPLC chromatogram attained from HPLC analysis of the 60% ethanol extract of BPSP. Each of the identified stilbenoid compounds was then calculated according to the standard curve constructed previously (Chang et al. 2006). As estimated, the contents of *trans*-resveratrol (Res), *tans*-arachidin-1 (Ara-1), *trans*-arachidin-3 (Ara-3) and *trans*-isopentadienylresveratrol (IPD) were 0.2, 2.9, 51.7 and 87.6 mg/g BPSP. The total stilbenoid content was ca. 142 mg/g BPSP and different levels of treatment diet were formulated by the specified stilbenoid level per kg body weight basis. As determined preliminarily, crude protein content of BPSP was equivalent to that of soybean protein. Accordingly, the soybean protein in the basal diet was replaced by BPSP (Table 1) except for the formulation of the super-high BPSP group where 10 g soybean protein and 15 g full-fat soybean powder were replaced by BPSP.

#### Food toxicological evaluation

An acute toxicity animal study was conducted to accommodate the guidelines of the 28-days feeding toxicity assessments for health food of the Taiwan Food and Drug Administration, the Ministry of Health and Welfare, Executive Yean, Taiwan. Proposal for the animal experimentation was approved by the Institutional Animal Care and Use Committee of National Chiayi University (NCYU-IACUC 2,006,005). A total of 48, 8-week-old ICR mice were obtained from BioLASCO Taiwan Co. (Taipei, Taiwan). Six mice of each gender were randomly assigned into 4 experimental groups and 3 mice of the same gender per cage. Animals were weighed and ear-tagged during the acclimation. The mice were housed in SPF facility with constant room temperature at 22 °C, 50% relative humidity and 12 h light/dark cycle. Experimental diet compositions were described above and shown in Table 1. The experimental treatment level of calculated stilbenoid content was justified by the daily feed consumption with an initial average daily feed intake of 4.5 g/male and 4.2 g/female mouse for the first week of acclimation. After feeding with basal diet (Table 1) for adaptation in the initial 2 weeks, the average body weights for male and female mice were 40 and 37 g, respectively. As affected by body weight and gender difference, the average daily feed consumption was 4.5 and 4.2 g for male and female mouse, respectively. The dietary treatments for daily feeding levels were 0 g (control group), 0.11 g (normal group), 2.5 g (high group) and 25 g BPSP/kg BW (super-high group) which were correspondingly equivalent to 0, 15, 350 and 3,500 mg stilbenoids/kg BW. The daily provided diets for the male and female cages were initiated by 13.5 and 12.6 g, respectively. Feeding diet quantity was adjusted weekly based on total weight increase for 3 mice in a cage, i.e., 0.12 g feed per gram body weight. Water was accessed ad libitum. During the entire 35 days of experiment, individual body weight was weighed weekly and daily health monitoring was proceeded as a routine practice.

#### Toxicological assessment

Animals were fasted overnight before euthanasia with CO_2_. Blood samples were collected with EDTA (K_2_ EDTA syringes, BD Vacutainer, Franklin Lakes, NJ, USA) tube via heart puncture immediately after euthanizing. Toxicological analyses were performed according to Weng et al. (2011) with slight modifications. An aliquot of whole blood sample was subjected to a hematological analyzer (Sysmex XE2100, TOA Medical Electronics, Kobe, Japan) including red blood cell (RBC), white blood cell (WBC), hemoglobin (HGB) and hematocrit (HCT). The remaining blood was centrifuged at 1,700 *g* for 15 min for plasma separation. Plasma samples were stored at -20 °C for biochemical analyses which were performed by an automated clinical chemistry analyzer (Abbott Architect Plus ci4100, Abbott, Abbott Park, IL, USA) operated by Sin-Lin Medical Laboratory Center, Chiayi, Taiwan. The determined items included aspartate transaminase (AST), alanine transaminase (ALT), total protein, blood urea nitrogen (BUN), cholesterol (T-chol), triglyceride (TG) and lactate dehydrogenase (LDH).

#### Autopsy and histopathological examination

Overall pathological examinations were performed after dissection. Major organs including the liver, kidney, spleen, heart and lung were removed and weighed. Organ index (relative organ weight) was obtained with the justification of body weight. Subsequently, the organs were then immersed in 37% neutral formaldehyde (Thermal Fisher Scientific, Inc., Waltham, MA, USA) for later histopathological section preparation. Briefly, formaldehyde fixed organs were delivered to tissue section specimen to be embedded in paraffin. Tissue block sliced into 10-µm sections were layered onto glass slides and subsequently stained with haematoxylin and eosin. Each specimen was then photographed by a CCD equipped microscope (Olympus IX71 and C-5050, Olympus Optical Co. Ltd., Tokyo, Japan) for histopathological examination by a veterinary pathologist.

### Statistics

Data were expressed as means ± standard deviation and analyzed using GLM model procedure (SAS Institute 1996). Significant differences (*P*-value < 0.05) among treatments were determined using Duncan’s New Multiple Range Test.

## Results and discussion

### Allergenicity evaluation

Soluble protein contents of the defatted cotyledon powders derived raw kernels, 72 h-NGS and BPSP were extracted with phosphate buffer saline (PBS). The soluble protein contents of the raw kernel, 72 h-NGS and BPSP were 3.74 ± 0.1, 3.4 ± 0.1 and 8.9 ± 0.1 mg/mL, respectively. In comparisons, raw kernels underwent germination for 72 h had a minor decrease on cotyledon soluble protein content. Chiou et al. (1997) have reported that three different cultivars of peanut kernel were subjected to germinate for 96 h had slightly reduced soluble proteins, while significant molecular size changes showed in SDS − PAGE patterns. Similar outcomes in changes of protein contents were also demonstrated by Kang et al. (2007), who reported that only a few percent reduction of soluble protein in fresh peanut cotyledons after germination for 96 h. Interestingly, the BPSP had an approximate 2.6-fold higher soluble protein content than that of 72 h-NGS. This indicates the bio-elicitation process may promote extensive proteolysis of the cotyledon storage proteins and result in increased soluble protein content.

**Fig. 1:**
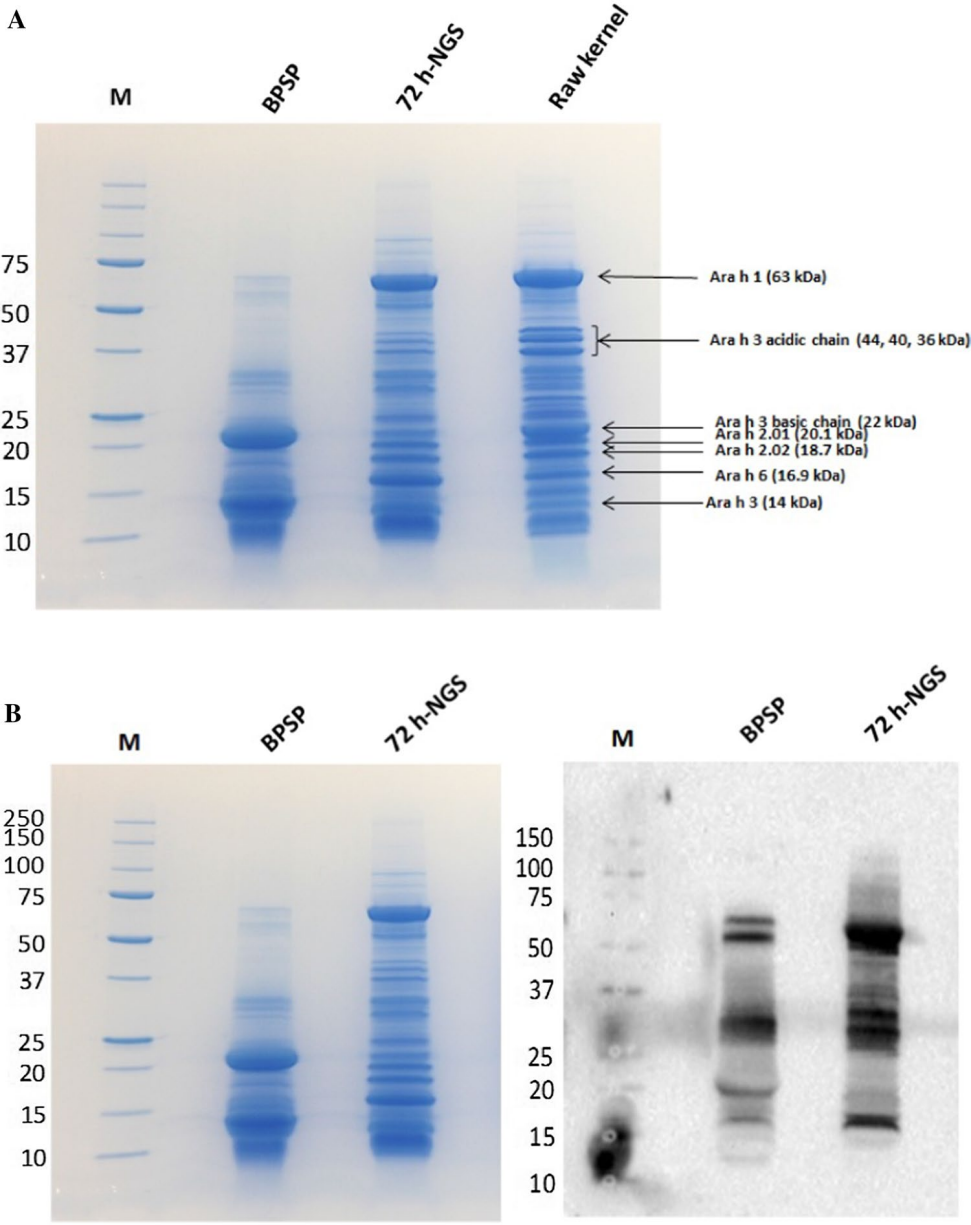
Approximately 17 µg of protein was loaded per lane. SDS-PAGE protein electrophoresis patterns indicated with arrows were known peanut allergens of soluble protein extracted from the defatted cotyledon powders of raw peanut kernel (Raw kernel), 72 h-germinated normal sprouts (72 h-NGS) and bio-elicited peanut sprout powder (BPSP) (**A**). Paralleled comparison of bio-elicited peanut sprout powder BPSP and 72 h-NGS on the specific protein degradation was demonstrated with SDS-PAGE analysis (Left) and followed immunoblotting detected with peanut allergic IgE containing serum (right). Protein degradation and diminishing of allergic proteins of size from 15 to 75 kDa were observed in BPSP as compared to 72 h-NGS (**B**)

SDS-PAGE protein patterns of soluble proteins demonstrated the major peanut allergens, Ara h 1, 2, and 3, were present in the raw kernels and 72 h-NGS (Fig. 1A). Based on immunoblot result (Fig. 1B), pooled IgE serum from peanut-allergic patients bound across molecular weights range from 25 to 75 kDa of the major peanut allergens in 72 h-NGS, while the bound allergens in BPSP were reduced. Georgia Green peanut seeds germinated for 72 h demonstrated a similar decreased contents of major peanut allergens (Kang et al. 2007). Although the current result did not demonstrate reduction of prominent bands over the 72 h germination (72 h -NGS), the combination of germination and the bio-elicitation procedures resulted in extensive degradation of the specific allergic proteins including Ara h 1 (63 kDa) and Ara h 3 (44, 40 and 36 kDa). As paralleled comparison to the bands of specific allergen Ara h 3, the Ara h 3 basic chain (22 kDa) and Ara h 3 (14 kDa) were remarkably increased whereas the Ara h 2.01 (18.7 kDa) and Ara h 2.02 (16.9 kDa) allergens decreased in BPSP (Fig. 1B left). Moreover, Ara h 6 (16.9 kDa) increased in 72 h-NGS as the prominent band seen in the gel of IgE-immunoblotting (Fig. 1B right), and diminished remarkably in BPSP. With the advance of molecular techniques, historical definitions of primary peanut allergens can be better realized by allergic effectors. Ara h 2 and Ara h 6 bear tertiary structure similarity and exhibit most allergic reactivity (Zhuang and Dreskin 2013). According to Koppelman et al. (2001), the major peanut allergens, namely, Ara h 1, Ara h 2, and Ara h 3, are seed storage proteins and responsible for more than 50% of peanut-allergic patients (Koppelman et al. 2001). As stated in earlier investigations, 70 to 90% of the peanut allergic patients are caused by Ara h 1 and Ara h 2 (Burks et al. 1995; Clarke et al. 1998). Accordingly, significant diminishment of the major peanut allergenic proteins during germination and the bio-elicitation processes could reduce reactivity.

Furthermore, it is noticeable that allergic patient’s IgE had a high affinity to proteins sized between 25 to 75 kDa as detected in 72 h-NGS were greatly reduced while the small sized IgE bound proteins (< 25 kDa) were still detected in BPSP (Fig. 1B right). In particular, the detected protein band around 20 kDa might be Ara h 2.01 and/or Ara h 2.02 that showed stronger IgE binding affinity in BPSP than that of 72 h-NGS. In BPSP, Ara h 1 was visualized as a doublet rather than a singlet band observed in the 72 h-NGS. This is also very likely due to degradation of the large molecular proteins during germination. Additionally, a remarkable reduction from two adjacent bands located at the size of Ara h 6 with a smaller molecular size was obtained. The subjection of peanut flour extracts to enzymatic hydrolysis has resulted in allergen degradation and changes of allergenic properties (Shi et al. 2012). From the point of view in development of BPSP-containing foods, the potential allergenic reductions observed in this study for BPSP give merit to further pursuit of BPSP as a novel food ingredient.

Conventionally, enzymatic hydrolysis seems to be one of the most advantageous techniques in diminishing the allergenic potential of peanut, since peanut proteins and allergens are not eliminated and do not evaporate upon thermal or vacuum-facilitated treatments (Cabanillas et al. 2012; Shi et al. 2012; White et al. 2014; Shah et al. 2019). As observed in this study, the indigenous proteases were most likely biosynthesized and functioning extensive enzymatic protein degradation during kernel germination and followed bio-elicitation. Based on temporal and spatial gene expressions of *ara h 1*, *ara h 2* and *ara h 3* were down regulated during peanut seed germination and seedling growth (Kang et al. 2007), transcripts of *ara h 3* were undetectable during seed germination. The levels of Ara h 1 and Ara h 2 dramatically reduced compared to the Ara h 3 polypeptides in embryonic axes as compared with cotyledons during germination and seedling growth. In this study, based on SDS-PAGE analysis of the soluble proteins extracted from the cotyledons, the protein patterns varied limitedly after 72 h of germination but changed substantially in the followed bio-elicitation treatment. Furthermore, immunoblotting results demonstrated remarkable reduction on peanut allergic patients’ IgE binding affinity in BPSP. The slicing and bio-eliciting processes might have initiated defense mechanism in generating secondary metabolites. Nevertheless, the reduction of allergenicity in BPSP demands further intensive investigations for food allergy and safety to facilitate future utilization in development of functional foods and nutraceuticals.

### Food toxicological assessment

In the 35-day dietary intervention study, the body weight changes insignificantly in male and female mice among treatment groups (supplemental materials). The average body weights remained relatively steady throughout the entire study. As further addressed on the super-high groups after feeding BPSP-supplemented diets formulated at 25 g BPSP/kg BW (equivalent to 3,500 mg stilbenoids /kg BW) for the entire 35 days, there was no sign of acute toxicity and all animals appeared sound and healthy. Thus, the maximum flexible range in incorporation of BPSP warrantees the safety threshold allowing a broad spectrum for supplementation in the related products development.

### Hematological and serum biochemical analyses

Blood samples were withdrawn from the mice at the end point after various levels of BPSP-dietary intervention were subjected to hematological and biochemical analysis and the results are shown in Tables 2 and 3. The values including WBC, RBC, HGB and HCT for both genders of ICR mice were all distributing in the referenced normal range of healthy ICR mice (Liang et al. 1999) and there was no significant difference among the treatment groups.

**Table 2:** Hematological and blood biochemical analysis of male ICR mice fed diets with different levels of bio-elicited peanut sprout powder

Treatment group (BPSP dose)/determinations^1,3,4^	Control (0 g/kg BW)	Normal BPSP (0.11 g/kg BW)	High BPSP (2.5 g/kg BW)	Super-high BPSP (25 g/kg BW)
Hematological analysis^2^				
WBC (10^3^/μL)	5.36 ± 3.01^a^	7.17 ± 4.60^a^	5.70 ± 3.78^a^	4.84 ± 2.56^a^
RBC (10^6^/μL)	9.52 ± 0.66^a^	9.41 ± 1.51^a^	8.13 ± 2.26^a^	9.12 ± 0.99^a^
HGB (mg/dL)	14.76 ± 1.31^a^	14.42 ± 2.34^a^	12.84 ± 4.12^a^	13.86 ± 1.50^a^
HCT (%)	49.88 ± 5.10^a^	48.62 ± 8.82^a^	42.90 ± 12.4^a^	46.74 ± 4.41^a^
Blood biochemical analysis^2^				
AST (U/L)	74.60 ± 24.46^a^	165.67 ± 101.64^a^	127.40 ± 47.43^a^	171.50 ± 114.39^a^
ALT (U/L)	63.00 ± 85.64^a^	79.17 ± 43.69^a^	51.20 ± 35.99^a^	39.00 ± 12.12^a^
T-P g/dL)	5.68 ± 0.47^a^	5.23 ± 0.80^a^	5.54 ± 0.24^a^	5.18 ± 0.49^a^
BUN (mg/dL)	27.86 ± 4.20^a^	29.35 ± 17.65^a^	23.62 ± 3.28^a^	27.61 ± 3.71^a^
T-Chol (mg/dL)	177.00 ± 25.81^a^	132.17 ± 52.07^a^	159.60 ± 38.87^a^	156.83 ± 47.38^a^
TG (mg/dL)	73.80 ± 17.03^a^	58.50 ± 43.71^a^	61.80 ± 50.73^a^	100.33 ± 41.24^a^
LDH (U/L)	1023.60 ± 703.23^a^	1108.17 ± 795.96^a^	833.20 ± 150.73^a^	1022.17 ± 472.46^a^

^1^Data are expressed as mean ± SD (n = 6)

^2^WBC: White blood count; RBC: Red blood cell; HGB: hemoglobin; Hct: Hematocrit; AST: Aspartate aminotransferase; ALT: Alanine aminotransferase; T-P: Total protein; BUN: Blood urea nitrogen; T-Chol: Total cholesterol; TG: Triglyceride; LDH: Lactate dehydrogenase

^3^Different superscript letter indicates significant difference between control (0 g BPSP/kg) and treated group at *P* < 0.05

^4^Normal range: WBC 6 ~ 15 × 10^3^ cell/μL; RBC 7 ~ 12.5 × 10.^6^ cell/μL; HGB 10.2 ~ 16.6 mg/dL; HCT 39 ~ 49%; AST 59 ~ 247 U/L; ALT 28 ~ 132 U/L; T-P 3.5 ~ 7.2 g/dL; BUN 17 ~ 28 mg/dL; T-Chol 26 ~ 82 mg/dL; LDH 159 ~ 1045 U/L

**Table 3:** Hematological and blood biochemical analysis of female ICR mice fed diets with different levels of bio-elicited peanut sprout powder

Treatment group (BPSP dose) /determinations^1,3,4^	Control (0 g/kg BW)	Normal BPSP (0.11 g/kg BW)	High BPSP (2.5 g/kg BW)	Super-high BPSP (25 g/kg BW)
Hematological analysis^2^				
WBC (10^3^/μL)	6.43 ± 2.15^a^	6.78 ± 3.18^a^	4.23 ± 2.27^a^	4.85 ± 1.55^a^
RBC (10^6^/μL)	9.86 ± 0.71^a^	9.40 ± 0.86^a^	9.06 ± 1.92^a^	8.60 ± 1.72^a^
HGB (mg/dL)	16.07 ± 0.67^a^	15.20 ± 1.32^a^	14.30 ± 2.78^a^	13.65 ± 2.72^a^
HCT (%)	53.42 ± 3.94^a^	49.80 ± 6.12^a^	46.12 ± 10.03^a^	44.67 ± 9.23^a^
Blood biochemical analysis^2^				
AST (U/L)	141.00 ± 47.07^a^	182.33 ± 134.84^a^	221.67 ± 151.28^a^	182.83 ± 101.41^a^
ALT (U/L)	49.83 ± 12.91^a^	48.50 ± 25.68^a^	84.17 ± 62.21^a^	52.00 ± 20.19^a^
T-P g/dL)	5.72 ± 0.23^a^	4.97 ± 0.93^a^	5.23 ± 0.59^a^	5.64 ± 0.43^a^
BUN (mg/dL)	19.05 ± 2.89^a^	23.40 ± 13.68^a^	19.37 ± 2.70^a^	22.02 ± 4.69^a^
T-Chol (mg/dL)	109.5 ± 30.76^a^	99.33 ± 22.42^a^	86.83 ± 10.78^a^	90.67 ± 13.78^a^
TG (mg/dL)	27.50 ± 10.29^a^	26.33 ± 13.87^a^	27.17 ± 21.62^a^	16.67 ± 4.68^a^
LDH (U/L)	717.67 ± 156.33^a^	796.33 ± 495.70^a^	1041.0 ± 641.73^a^	843.17 ± 372.86^a^

^1^Determinations are expressed as mean ± SD (n = 6)

^2^WBC: White blood count; RBC: Red blood cell; HGB: hemoglobin; Hct: Hematocrit; AST: Aspartate aminotransferase; ALT: Alanine aminotransferase; T-P: Total protein; BUN: Blood urea nitrogen; T-Chol: Total cholesterol; TG: Triglyceride; LDH: Lactate dehydrogenase

^3^Different superscript letter indicates significant difference between control (0 g BPSP/kg) and treated group at *P* < 0.05

^4^Normal range: WBC 6 ~ 15 × 10^3^ cell/μL; RBC 7 ~ 12.5 × 10.^6^ cell/μL; HGB 10.2 ~ 16.6 mg/dL; HCT 39 ~ 49%; AST 59 ~ 247 U/L; ALT 28 ~ 132 U/L; T-P 3.5 ~ 7.2 g/dL; BUN 17 ~ 28 mg/dL; T-Chol 26 ~ 82 mg/dL; LDH 159 ~ 1045 U/L

Serum biochemical analysis (Tables 2 and 3) including aspartate transaminase (AST), alanine transaminase (ALT), total protein (TP), urea nitrogen (BUN), total cholesterol (T-Chol), triglyceride (TG) and lactate dehydrogenase (LDH) for the experimental groups of both genders as affected by BPSP supplementation was conducted. All values were distributed in the reference normal ranges of healthy mice and there was no significant difference among test groups (*P* > 0.05). Based on the current data, there was no obvious health hazard in the BPSP-fed mice. Aspartate transaminase (AST) and alanine transaminase (ALT) level in plasma reflect liver function, all determined values distributed in the normal mouse ranges of 59–247 U/L for AST and 28–132 U/L for ALT, respectively (Liang et al. 1999). Based on the determined BUN values, there was no obvious hazard for kidneys as affected by BPSP-supplemented diets. In addition, the determined values of T-Chol, TG and LDH were also distributing in the normal ranges of which 26–82 mg/dL for T-Chol, 20–80 mg/dL for TG and 159–1045 U/L for LDH, respectively (Liang et al. 1999). All serum biochemical determinations warrantee toxicological acceptance of the diets supplemented BPSP from low to super high levels.

### Relative organ weight and histopathological examination

The relative weights of liver, kidney, spleen, heart and lungs of body weights are shown in Table 4. Dietary supplementation with various levels of BPSP on the relative organ weights were not significantly different among groups and between genders, except relative liver weight of the male mice fed with the super-high dose of BPSP (25 g/kg BW, equivalent to 3,500 mg stilbenoids/kg BW) which was significantly higher (*P* < 0.05) than the control group. With reference to the correlated serum biochemical determinations including AST, ALT, TP, BUN, T-Chol, TG and LDH (Tables 2 and 3), all values were distributed in the reference normal ranges of healthy mice and there was no significant difference among test groups (*P* > 0.05). For the super-high diet formulation, BPSP was used in substitution of all soy protein and part of the full-fat soybean powder in the basal diet (Table 1). The dietary uptake may cause metabolic loading burden and increase relative liver weights for the male mice.

**Table 4:** Relative organ weights of ICR mice fed diets with different levels of bio-elicited peanut sprout powder

Treatment group (BPSP dose)	Control (0 g/kg BW)	Normal BPSP (0.11 g/kg BW)	High BPSP (2.5 g/kg BW)	Super-high BPSP (25 g/kg BW)
Male	Relative organ weight (%)^1^^,2,3^			
Liver	3.599 ± 0.386^b^	3.730 ± 0.317^ab^	4.055 ± 0.372^ab^	4.266 ± 0.416^a^
Kidney	1.473 ± 0.240^a^	1.078 ± 0.073^a^	1.670 ± 0.150^a^	1.535 ± 0.182^a^
Spleen	0.328 ± 0.103^a^	0.385 ± 0.095^a^	0.440 ± 0.173^a^	0.441 ± 0.085^a^
Heart	0.444 ± 0.068^a^	0.497 ± 0.061^a^	0.459 ± 0.047^a^	0.430 ± 0.034^a^
Lung	0.623 ± 0.187^a^	0.604 ± 0.187^a^	0.718 ± 0.208^a^	0.606 ± 0.069^a^
Body weight, g^4^	39.12 ± 2.16^a^	35.69 ± 4.41^a^	36.04 ± 3.48^a^	39.06 ± 2.75^a^
Female	Relative organ weight (%)			
Liver	3.773 ± 0.408^a^	3.830 ± 0.290^a^	3.874 ± 0.156^a^	4.000 ± 0.369^a^
Kidney	1.486 ± 0.147^a^	1.486 ± 0.078^a^	1.471 ± 0.162^a^	1.410 ± 0.126^a^
Spleen	0.413 ± 0.143^a^	0.459 ± 0.074^a^	0.483 ± 0.141^a^	0.530 ± 0.084^a^
Heart	0.482 ± 0.047^a^	0.506 ± 0.045^a^	0.508 ± 0.047^a^	0.485 ± 0.076^a^
Lung	0.620 ± 0.068^a^	0.589 ± 0.140^a^	0.679 ± 0.098^a^	0.649 ± 0.061^a^
Body weight, g^4^	26.29 ± 1.88^a^	24.75 ± 2.20^a^	26.22 ± 2.80^a^	25.38 ± 2.74^a^

^1^Relative organ weight (%) = [organ weight (g)/body weight (g)] × 100

^2^Data are expressed as mean ± SD (n = 6)

^3^Different superscript letter indicates significant difference between control (0 g BPSP/kg) and treated group at *P* < 0.05

^4^Body weight was an average of accumulated measurements throughout the entire study. Change of body weight over the entire 35-day study was provided as supplemented materials

**Fig. 2:**
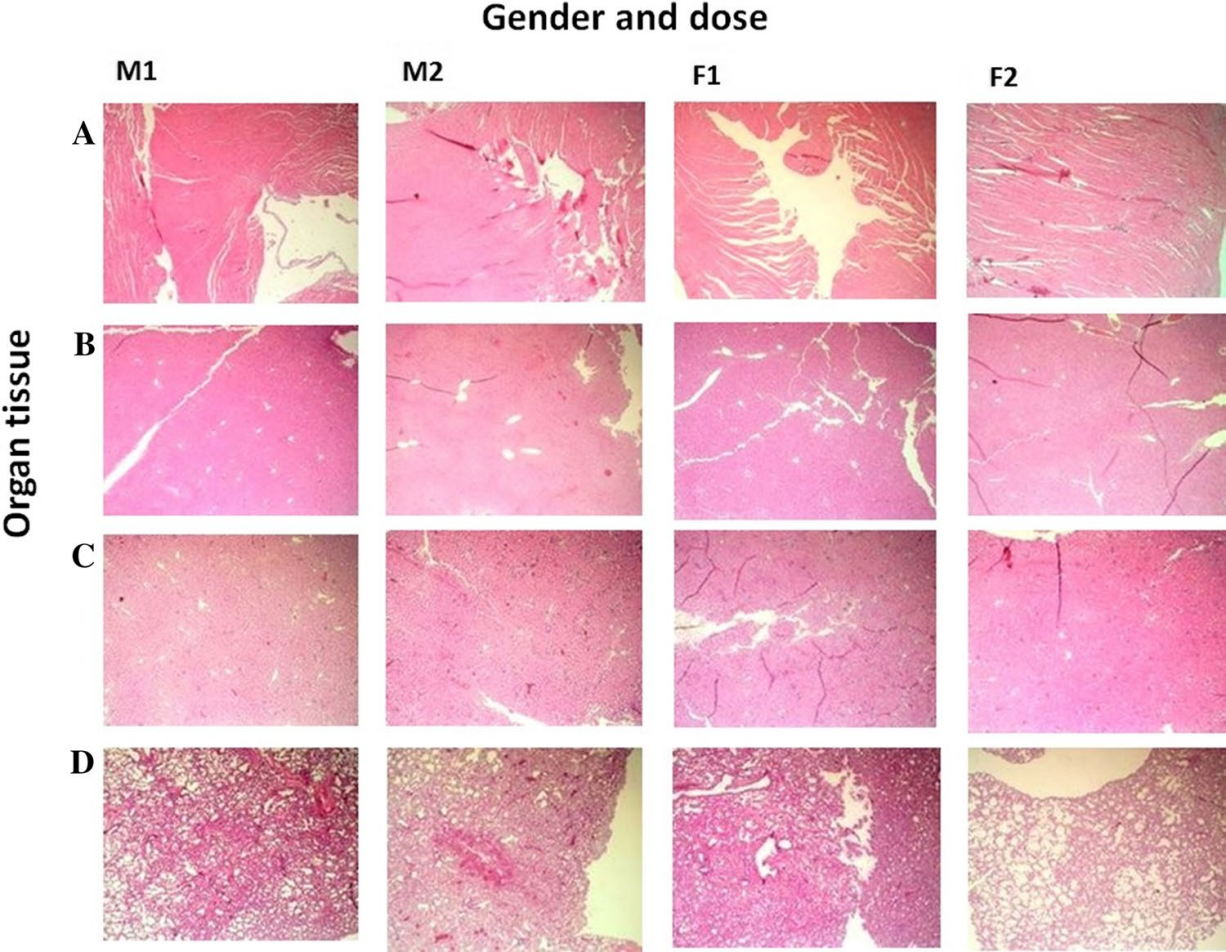
Histopathological examinations on heart (**A**), liver (**B**), kidney (**C**) and lung (**D**) of male (**M**) and female (**F**) mice fed with basal diet (**1**) with diet supplemented with super-high dose of BPSP (25 g BPSP/kg) (**2**) for 35 days

Histopathological examinations on the sections of heart, liver, kidney and lung tissues revealed no sign of abnormal or lesion of the major organs. The focal areas under microscopic examination are shown in Fig. 2. As addressed on relative liver weight exhibited dose-dependent increase and the super-high male group was significantly higher than that of the control group (Table 4), minor sign of fatty liver was noticed which was in agreement with our assumption that additional calories uptake from BPSP supplemented super-high diet may contribute to the lipid accumulation in liver and increased relative liver weight. Nevertheless, based on the results of relative organ weight and the histopathological examinations, there was no obvious sign of toxicity in the current dietary intervention of safety assessment on the BPSP.

Best food safety practices mandate the labeling of peanut allergen-contained ingredients used in food formulations and to indicate possible facility contact with peanut commodities to alert peanut allergenic individuals to avoid peanut allergens. However, occasional allergenic incidents are still inevitable and most adverse events occur with accident ingestion (Shah et al. 2019). While further study is needed, and total avoidance of peanut-based ingredients should always be stressed for allergic individuals, this study suggests accidental exposures of BPSP by allergic individuals should be no worse, or perhaps even milder, compared to traditional defatted peanut powders. Furthermore, this study showed there was no obvious health concern of mice fed with a broad spectrum of dose of BPSP-supplemented diets from 0.11 to 25 g BPSP/kg BW for 35 days. Similarly, our previous animal experiment with the Sprague–Dawley rats fed with dietary fresh peanut sprouts up to 16.5 g/day for 18 weeks (Lin et al. 2008) has sound health. Experiments respectively conducted with aged BALB/c and ICR mice BPSP-supplemented diets daily for 750 and 762 days in the doses of 0.1 and 0.5 g BPSP/kg BW had average ca. 30% extension on lifespan longer than the mock groups (Chiou et al. 2017). Accordingly, the reduced allergenicity and food toxicological acceptance of BPSP gives merit to pursuing BPSP as a novel food ingredient.

## Conclusion

In this study, it was demonstrated that specific peanut allergic patients’ serum IgE binding in vitro was reduced in BPSP compared to appropriate controls. Further studies are needed to elucidate the mechanistic interaction of the bio-elicited synthesized metabolites and the alterations of allergic protein moieties leading to the potential allergenicity reduction of BPSP. Furthermore, there was no obvious acute health concerns of ICR mice fed with BPSP-supplemented diets. These promising food safety data give merit to pursuing more detailed applications for BPSP as a healthy, functional ingredient.

## Supplementary Information

Below is the link to the electronic supplementary material.

Supplementary file 1 (DOCX 60 kb)

## Data Availability

The original slides, images, and raw analytical data are considered to be available upon request or to be provided as supplementary materials.

## Abbreviations

72 h-NGS: Peanuts: 72 h normally germinated sprouts
BPSP: Bio-elicited peanut sprout powder

## Code availability

Our labs in the university basis are under the guidance of Good Laboratory Practice, all chemicals and consumables were purchased and prepared with MSDS and according to the manufacture recommendations. BPSP was prepared in three different batches with a standard operation procedure. Experimental diets were freshly made with inclusion of different amounts of BPSP supplementation at doses of 0, 0.11, 2.5 and 25 g BPSP/kg BW. Sample of BPSP is ready for interest investigators and collaborators upon request.
