# Supplementary material for: Allergenicity reduction of the bio-elicited peanut sprout powder (BPSP) and toxicological acceptance of BPSP-supplemented diets assessed with ICR mice
Source: J Food Sci Technol. 2022 Jul 10;59(12):4583–93. doi: 10.1007/s13197-022-05537-7 (PMC9579254; doi:10.1007/s13197-022-05537-7)
Supplement: Supplementary file 1 — (DOCX 60 kb) [file 13197_2022_5537_MOESM1_ESM.docx]

**MS (JFST-D-21-01971R1): Allergenicity reduction of the bio-elicited peanut sprout powder (BPSP) and toxicological acceptance of BPSP-supplemented diets assessed with ICR mice**

**Supplementar Material:**

**Figure 1**


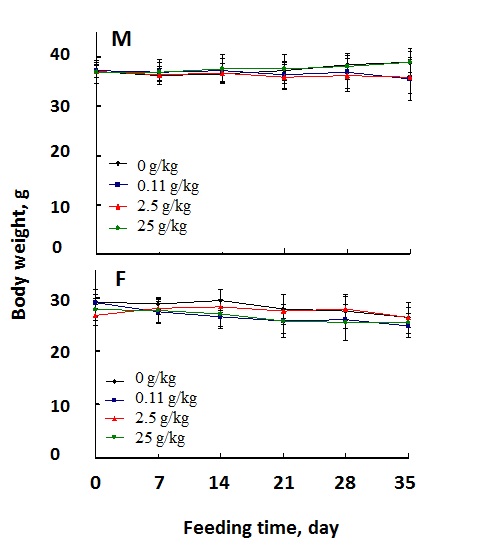


**Supplement Figure 1.** Body weight changes of ICR male (M) and female (F) mice fed with BPSP-supplemented diets for 35 days.
